# Retinotopic effects of visual attention revealed by dichoptic multifocal pupillography

**DOI:** 10.1038/s41598-018-21196-1

**Published:** 2018-02-14

**Authors:** Yanti Rosli, Corinne Frances Carle, Yiling Ho, Andrew Charles James, Maria Kolic, Emilie Marie Françoise Rohan, Ted Maddess

**Affiliations:** 1 Eccles Institute of Neuroscience, John Curtin School of Medical Research, Australian National University, Canberra, Australia; 2 Biomedical Science Program, Diagnostic & Applied Health Sciences, Faculty of Health Science, Universiti Kebangsaan Malaysia, Jalan Raja Muda Abdul Aziz, 50300 Kuala Lumpur, Malaysia; 3 Centre for Eye Research Australia, University of Melbourne, East Melbourne, Australia

## Abstract

Multifocal pupillographic objective perimetry (mfPOP) has recently been shown to be able to measure cortical function. Here we assessed 44 regions of the central 60 degrees of the visual fields of each eye concurrently in 7 minutes/test. We examined how foveally- and peripherally-directed attention changed response sensitivity and delay across the 44 visual field locations/eye. Four experiments were completed comparing *white, yellow* and *blue* stimulus arrays. Experiments 1 to 4 tested 16, 23, 9 and 6 subjects, 49/54 being unique. Experiment 1, Experiments 2 and 3, and Experiment 4 used three variants of the mfPOP method that provided increasingly improved signal quality. Experiments 1 to 3 examined centrally directed attention, and Experiment 4 compared effects of attention directed to different peripheral targets. Attention reduced the sensitivity of the peripheral locations in Experiment 1, *but only for the white stimuli not yellow*. Experiment 2 confirmed that result. Experiment 3 showed that blue stimuli behaved like white. Peripheral attention showed increased sensitivity around the attentional targets. The results are discussed in terms of the cortical inputs to the pupillary system. The results agree with those from multifocal and other fMRI and VEP studies. mfPOP may be a useful adjunct to those methods.

## Introduction

The study of visual attention has grown prodigiously over the past 25 years. Although there is some evidence of increased sensitivity to attended items, behavioural studies have generally indicated that there is suppression of sensitivity peripheral to the focus of attention^1–4^. In seeming agreement early fMRI studies of attention reported reduced BOLD signals for unattended parts of the visual field^5–9^. Recent studies indicate that these changes are not due to blood stealing^10^, and that they are correlated with independent measures of reduced brain responsivity^11–15^.

The suppressive effects are best seen when several peripherally placed distractors are presented together. A genre of experimental methods where this occurs as a matter of course are the *multifocal* methods, where responses are obtained to many independent stimuli that are concurrently presented across the visual field. For such stimuli, there is scope for attended and unattended stimuli amongst the concurrently presented ensemble, as can occur in nature. Attention has been reported to enhance multifocal VEP (mfVEP) responses at the attended location, and also to produce a mixture of milder enhancement and suppression at adjacent unattended locations^16^. Those patterns of sensitivity change are similar to those reported for fMRI methods that use multifocal-like methods^17,18^. One of us has extended multifocal methods to fMRI assessing retinotopic brain mapping^19,20^. That multifocal fMRI method has been applied to visual attention showing focal enhancement and peripheral suppression^21^. Here we describe a convenient and lower-cost multifocal method with which to explore attentional effects across the visual field: *multifocal pupillographic objective perimetry* (mfPOP).

Early versions of mfPOP have produced good diagnostic performance for glaucoma^22–24^, macular degeneration^25,26^, and early-stage diabetic retinopathy^27,28^. More recently the method has shown clinically useful results in multiple sclerosis^29^. The pupillary system is known to receive input from at least 7 extra-striate visual cortical areas^30^. Two of our recent studies confirm cortical input to mfPOP responses^24,31^. Furthermore, by using combined mfPOP and mfVEPs, we have recently verified extra-striate cortical drive of the irises in 42 subjects for stimuli like those used here^33^. A recent fMRI study reported that extra-striate cortical areas beyond V4 have been shown to have wider zones of peripheral suppression extending to 10 to 20 degrees away from the focus of attention^18^, well within the parts of the visual field tested by the mfPOP stimuli used here.

Here we mainly examine whether a foveally-directed attentional task suppresses mfPOP responses to ensembles of 44 multifocal stimuli per eye, presented at locations spanning 1 to 30 degrees eccentricity. This study arose as part of an investigation of mfPOP responses to stimulus ensembles of yellow or white stimuli presented at a range of luminance levels^33^. In our initial experiments on 16 subjects, the results for centrally directed attention were counter-intuitive: responses to peripherally presented white stimuli were suppressed, but this was not seen for the yellow stimuli. We therefore repeated the experiment with 23 different subjects, different experimenters, and mfPOP stimuli that had improved signal to noise ratios. Since white stimuli differ from yellow in containing blue we also examined blue *vs*. yellow stimuli in a further 9 subjects. Finally we compared the effects of attention directed to peripheral targets using the newest mfPOP stimulus methods^26^ on 6 subjects. We present the results of the four studies here and discuss them in terms of inputs to the pupillary system.

## Methods

### Subjects

Experiment 1 examined 16 volunteer participants (8 females; mean age 23.8 ± 7.1 SD years). Experiment 2 included 23 subjects (12 females, 39 ± 13.6 years). Experiment 3 examined 9 subjects (6 females, 40.4 ± 7.8 SD years). Experiment 4 tested 6 subjects (3 females, 39.8 ± 9.62 years). Subjects had normal or corrected visual acuity of 6/9 or better. Their visual fields were tested with the Humphrey FDT-C20 visual field test (Carl Zeiss Meditec, Dublin, CA), and none showed visual deficits. The study conformed to the Declaration of Helsinki and subjects gave written informed consent under the Australian National University’s Human Experimentation Ethics protocols 04/238 and 10/061. Subjects were requested to refrain from caffeine or alcohol at for least one hour before each session.

### Apparatus

**Figure 1:**
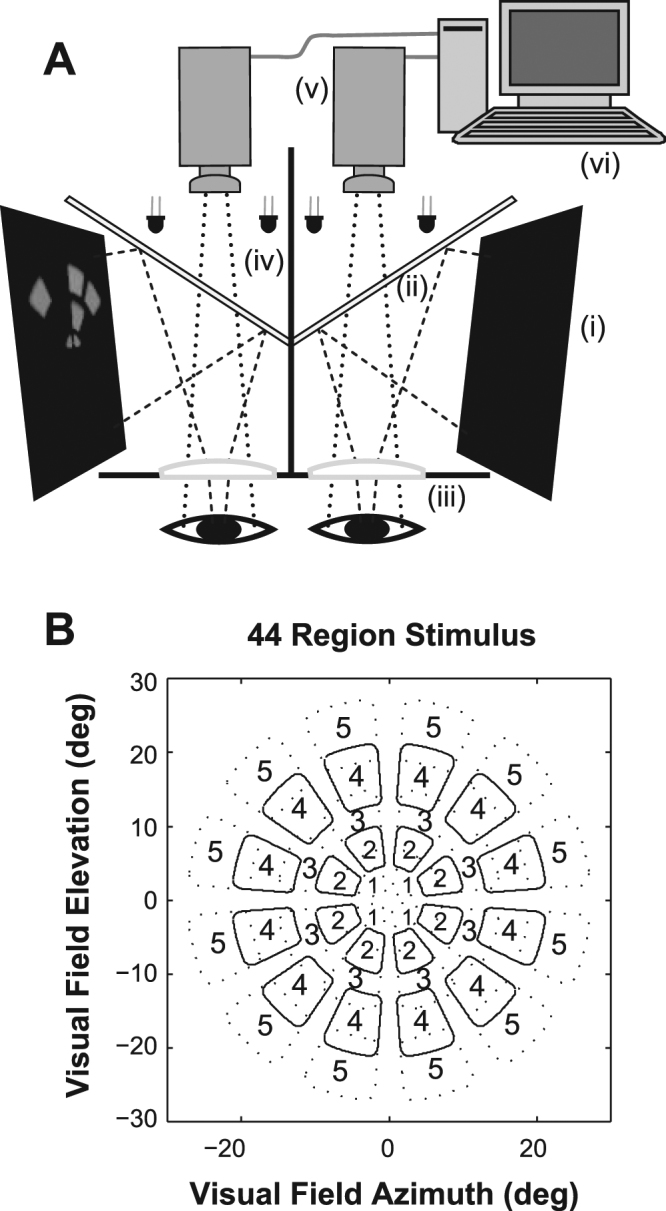
mfPOP Stimulator and Five-Ring Ensemble of Stimuli. (**A**) Stimuli are presented on two LCD displays (i) in a stereoscopic arrangement. Two dichroic mirrors (ii) direct the stimuli to the two eyes. Objective lenses (iii) render the stimuli at optical infinity. LEDs (iv) provide infrared (IR) illumination of the eyes. IR video cameras (v) image the pupils. The host computer (vi) tracks the pupil diameters at 60 frames/s synchronous with the displays. Eye positions and blinks are also recorded, and real-time feedback regarding the tracking accuracy is presented to the operator. (**B**) The individual stimuli have smoothly blurred sides and so are dominated by spatial frequencies <2 cpd. The contours shown represent the 80% level of the luminance of each region. The 5 concentric rings (numbers) of stimulus regions are somewhat overlapping to reduce under-sampling of the visual field. There is no overlap across the vertical and horizontal meridians. The fixation cross was 1.6 degrees across and was presented within the central 2 degrees of the visual fields.

Multifocal visual stimuli were presented using a prototype of the FDA-cleared nuCoria Field Analyzer (nCFA) (nuCoria, Acton, Australia), which records pupillary responses to dichoptic stimuli presented to both eyes (Fig. 1A). Refractive errors were corrected to the nearest 1.5 D. The nCFA stimuli are blurred so that they contain no spatial frequencies above 2 cpd, and thus refractive errors of <2 D do not significantly reduce the contrast of the stimuli. During the central attentional tasks, a button was provided for the subject to press (see Stimuli and Procedure below).

### Stimuli

The stimulus ensemble for each eye contained 44 test regions extending from 2 to 30 degrees eccentricity. The stimuli are arranged in 5 concentric rings (Fig. 1B). In Experiment 1 all the stimuli had the same luminance, 288 cd/m^2^, as was used in the original mfPOP methods^27^. The stimuli of Experiments 2 to 4 had slightly different luminances across the regions. These differences were introduced to balance out differences in visual field sensitivity^23^. The maximum luminance of any test stimulus was 150 cd/m^2^. Aside from yielding more similarly sized responses at each tested region this *luminance balancing* also improves the median signal to noise ratios obtained by about 15% in the least responsive regions of the visual field^23^. The stimuli of Experiment 4 used the *clustered volleys* method which improve signal to noise ratios by 40% relative to the presentation methods of Experiments 2 and 3^26^. Thus the experiments of this paper illustrate the evolution of the mfPOP method to date.

There were three colour-variants of the test stimuli: white, yellow and blue. The cone activations and CIE coordinates of the stimuli are given elsewhere^24,31^. All stimuli were presented against a background of 10 cd/m^2^. The temporal presentation of each of the stimulus regions was modulated by independent pseudo-random sequences. The transient onset stimuli were presented for a duration of 33 ms. In Experiment 1 the mean presentation interval was 1 s/region, generating 44 stimuli/s/eye; in Experiments 2 to 4 the mean interval was 4 s/region or 11 stimuli/s/eye. Those slower presentation rates have been shown to further improve signal to noise ratios^23^. In Experiment 1 each test was divided into eight segments of 30 seconds duration (4 minutes in total), whereas in Experiments 2 to 4 there were nine segments of 40 seconds (6 minutes). Rest periods of 7 seconds were given between segments. In the central Attention condition the centrally located fixation object changed transiently from a cross to a dot at random intervals during the course of each test sequence. This dot contained the same number of pixels as the cross. The fixation object remained a cross for the duration of the No-Attention condition. The fixation stimuli were red, were 1.6 deg square, and were presented within the central 2 deg of the stimulus ensembles. It is worth noting that in the No-Attention condition that the slow delivery of the transient onset stimuli meant that attention could not be distributed across a great number of stimuli at any one time. Experiment 4 employed a red 1.1 deg diameter circular attentional target displayed at either plus or minus10 deg eccentricity along the vertical midline. The mfPOP stimuli do not cross the horizontal of vertical meridians (Fig. 1)^24^, so the attentional targets did not overlap with any multifocal stimulus element. Subjects fixated centrally but attended to whichever target was presented.

### Procedure

In Experiment 1, the study was divided into two testing sessions, half the subjects first seeing the white stimuli in session 1, and half the yellow stimuli. Subjects were shown the other colour of stimuli in session 2. Subjects were randomized to doing or not doing the attentional task in one of the sessions. The task involved pressing the response button when they saw the fixation target change from the cross to the dot. In Experiments 2 and 3, all subjects were tested with four test protocols in a single session. For Experiment 2 these protocols presented: yellow stimuli with attention, yellow stimuli without attention, white stimuli with attention, and white stimuli without attention. In Experiment 3 transient blue stimuli were substituted for the white stimuli of Experiment 2. The order of these protocols was randomized across the subjects. Subjects were naïve as to the objectives of the two experiments. The experimenters were YH in Experiment 1, YR in Experiment 2,3, and CC and ER in Experiment 4.

Blinks or fixation losses during a test sequence were automatically labeled as invalid data^22,27^. If more than 15% of the data was lost from a particular test segment, it was repeated. Subjects were asked to blink several times prior to each test segment in order to minimize the blinking during the test. The multifocal analysis methods used in this experiment have been given in detail^22,27^. The regressive analysis method produced a standard error for each contraction amplitude and time to peak. These allow a t-statistic to be computed for each regional response amplitude and delay. The multifocal analysis is a form of multiple regression, so the standard errors are quite similar for each test region. The resulting t-statistics provided a measure of the signal reliability for each region, separate t-statistics being available for each region from each pupil.

### Statistics

The per region pupil constriction amplitudes in micrometers were transformed using a generalized log-transformation to stabilize the variance, and sensitivity at each visual field region was expressed in 10log_10_ decibels. To examine average regional effects, linear models were fitted that contained factors for region, and region by attention (Matlab ver 2016b). We also fitted effects for sex and consensual pupil response (response of a pupil to stimuli presented to the opposite eye). To examine the effect of eccentricity and deal with issues of multiple comparisons/subject, we fitted linear mixed-effects models. For Experiments 1 to 3 these were based on the mean response for each of the five rings of the stimuli (Fig. 1B), for each eye and pupil (R Foundation for Statistical Computing, version 3.2.0). These models contained effects for ring, ring by attention, sex and consensual pupil response. For Experiment 4 five analysis zones that pooled data for the left, inferior, right, superior and central field were substituted for rings. The central zone averaged results for the central 12 visual field regions, while the four peripheral zones averaged data from 8 visual field regions each.

### Data availability

The datasets generated during and/or analysed during the current study are available from the corresponding author on reasonable request.

## Results

### Experiment 1

**Figure 2:**
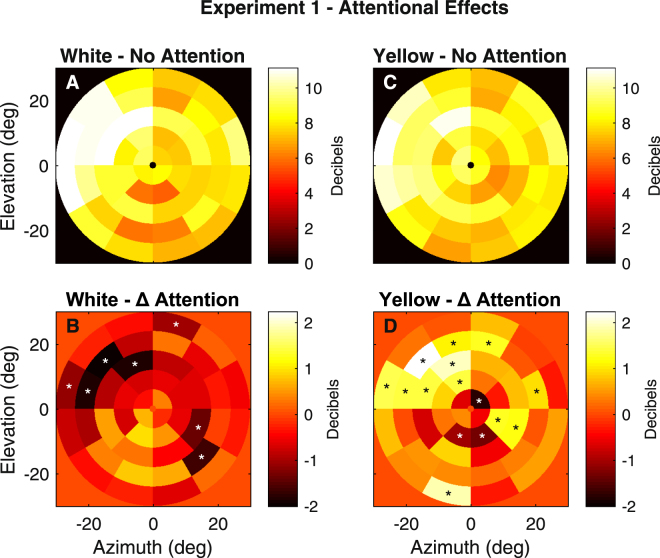
Regional Effects of Attention from Experiment 1. (**A**,**C**) The mean sensitivity of the 44 regions per eye obtained without the foveal attentional task for (**A**) white, and (**C**) yellow stimuli. Each region indicates the mean sensitivity computed across the 16 subjects, 2 pupils and 2 eyes. Before averaging, data from right eyes was flipped left to right so that they are left-eye equivalent, thus the temporal visual field is on the left. The mean responses were quite similar for white and yellow stimuli. (**B**,**D**) The mean effects (differences) produced by attention at each region (Δ Attention). The data are the terms of region by attention from the same linear models that generated the data of A and C. The medium orange backgrounds represent no effect (cf. calibration bars). Regions that on average are more or less responsive (lighter or darker tones) at the p < 0.05 level are tagged with a ‘*’. White stimuli appeared to generate peripheral suppression in response to attention. Yellow stimuli appeared to generate enhancement on average.

Figure 2 illustrates the average effects of the attentional task upon each visual field region. The four figures are derived from linear models (Methods). The upper two panels (Fig. 2A,C) give the mean sensitivity at each of the 44 visual field locations for the No-Attention condition. Thus, the luminance of each coloured region indicates the mean sensitivity computed across eyes, pupils, and subjects (N = 64 data sets). Prior to fitting the models, the responses for right eyes were reversed right to left, and so the data are all presented as for the left eye, with the temporal visual field on the left. Figure 2B,C are the region by attention effects, averaged across eyes, pupils, and subjects (Δ Attention). The regions with a ‘*’ indicate regions in the Attention condition that deviated from those of the No-Attention condition at the p < 0.05 level, the background pale orange indicating zero difference, darker red hues indicating suppression. The results seem to suggest that, on average, centrally directed attention caused peripheral suppression of the responses to the white stimuli (Fig. 2B). In the case of yellow stimuli 12 regions showed average enhancement of responses across the subjects, and three regions showed suppression (Fig. 2D).

### Experiment 2

**Figure 3:**
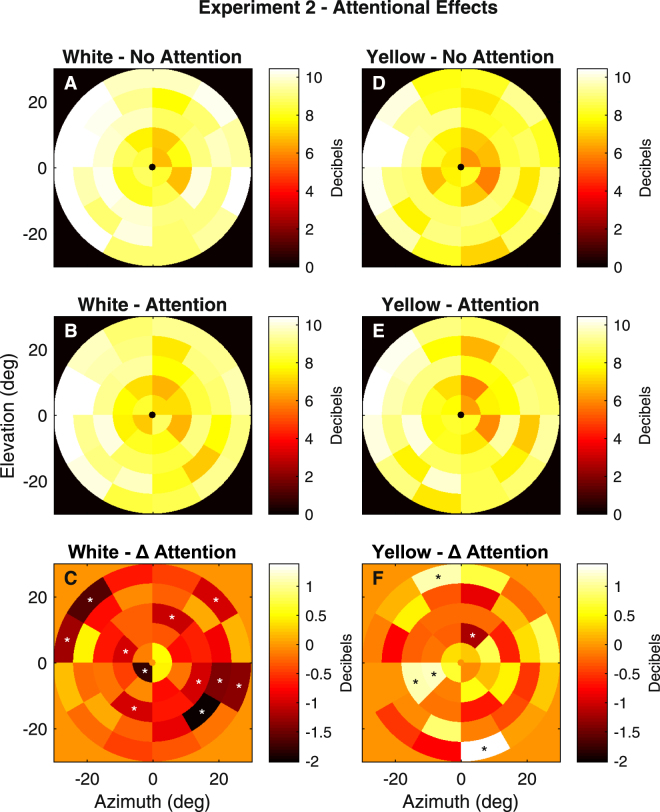
Regional Effects of Attention from Experiment 2. (**A**,**B**,**D**,**E**) The mean sensitivity for the No-Attention (AD), and Attention cases (BE) in the same subjects for the white (**A**,**B**), and yellow (**D**,**E**) stimuli. (**C**,**F**) The regional effects of attention, linear models and plotting conventions as in Fig. 2.

Following the initial result we created stimuli that were spatially similar but, due to their slower presentation rate and somewhat different luminances across the field, provided better signal to noise ratios^23^. We used these new stimuli to check the result of Experiment 1. In Experiment 2, all 23 subjects performed experiments with and without attention, for both white and yellow stimuli. Figure 3 illustrates the outcomes. The mean sensitivities obtained with and without attention are given in the top two rows (Fig. 3A,B,D,E) (N = 92 averaged fields/figure). The region by attention effects are given in Fig. 3C,F (Δ Attention). The results are broadly similar to Fig. 2, with 11 white stimulus regions producing suppression, 10 of them peripheral, while the yellow stimuli produced a mix of suppression and enhancement.

**Figure 4:**
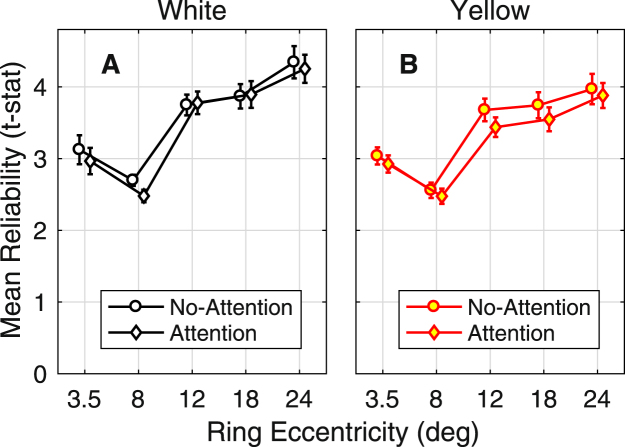
Signal reliability for Experiment 2 expressed as t-statistics (Mean ± SD). The regressive method for determining the response to the stimuli meant that each of the 176 sensitivity measurements/subject (2 eyes × 2 pupils × 44 regions/eye) has its own SE. Thus, a univariate t-statistic testing the case of no response could be computed, to quantify the reliability of the 176 pupil responses. The plotted values are the means around the 5 rings of the stimulus arrays for the 23 subjects of Experiment 2. The abscissa gives the eccentricity of the centre of each ring. The symbols for the No-Attention and Attention cases are slightly offset to the left and right to allow the symbols and error bars to be seen. The signal reliability was uniformly high in the peripheral regions where the effects of attention were most evident.

Figures 2 and 3 indicate some variability in the results. This variability might be due to sources such as: subject-wise issues, the particular attentional tasks, or noise. Figure 4 presents a measure of reliability of the mfPOP responses for the four conditions of Experiment 2 expressed as the mean (±SD) t-statistic for each of five rings of the stimuli (Methods). The t-statistics are calculated as the regional result divided by its SE. As mentioned in the Methods the multifocal analysis is essentially a multiple regression and so the SE for a given subject are fairly constant, hence variations in the t-statistic are mainly driven by changes in sensitivity or delay. For response amplitude, these t-statistics test the null hypothesis that there was no response. For the peripheral rings (Fig. 1B) the mean t-statistics were around 4 for all four test conditions indicating that the pupil signals were reliable.

### Experiment 3

Experiment 3 was similar to Experiment 2 except that blue transient stimuli were substituted for the white stimuli. Given that the yellow stimuli were trending towards enhancements with attention, then based on the idea that White = Yellow + Blue, one might expect the suppressive effects of attention might be somewhat larger for blue than for white. The analysis that follows indicates this is correct.

### Eccentricity analysis

We decided to address the issues of the average effect of attention as a function of visual field eccentricity. The average regional effects of attention as presented in Figs 2B,D and 3C,F are reported as in automated perimetry tests using the univariate t-statics at each location (produced by the multiple regression models), which might be considered problematic. We therefore used linear mixed-effects models that computed the mean suppression or enhancements for each of the five rings of stimuli. These models contained 20 measures per subject (5 rings × 2 eyes × 2 pupils), and the mixed-effects models accounted for these multiple measures/subject. The equivalent linear models were also computed for comparison (using the lmer and lm functions of the lme4 library of R). As in the linear models used to produce Figs 2 and 3 we included effects for sex, and consensual response. Correcting for multiple comparisons reduced the t-statistics by about 10%, suggesting that the ring-wise effects were substantially independent.

**Figure 5:**
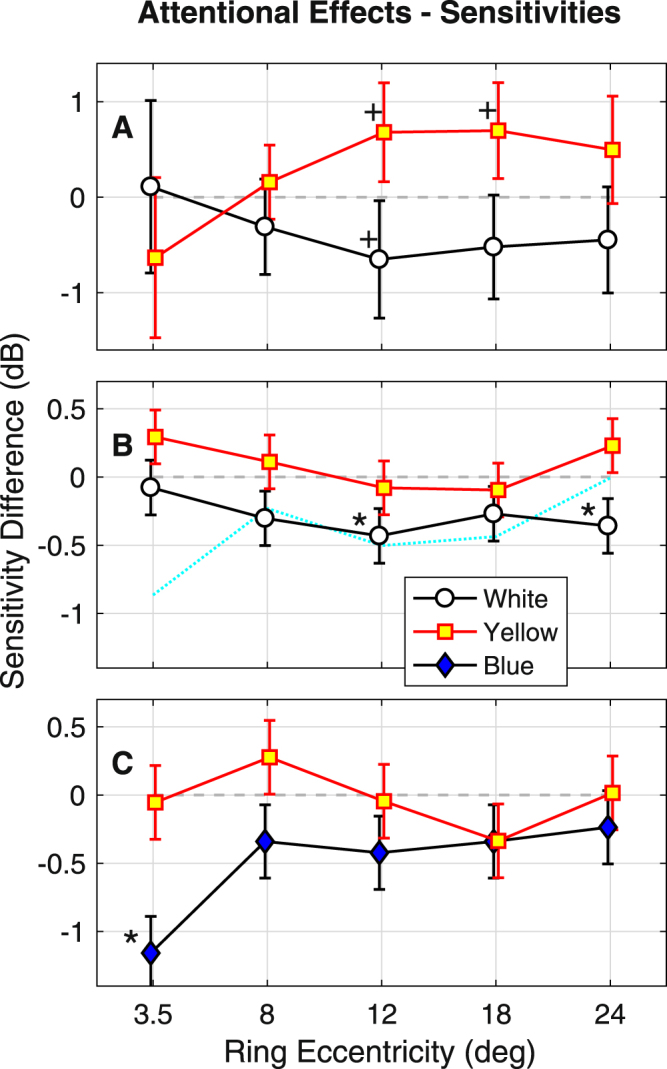
Effects of Attention upon sensitivity as a Function of Eccentricity. (**A**) Output of linear models for the data of Experiment 1 quantifying the region by ring effects. Separate models of the same form were computed for the white and yellow stimuli. The symbol face colors indicate the stimulus color (legend below). “+” Indicate significant differences at p < 0.05 for the linear models. (**B**) Output of a linear mixed-effects models for the white and yellow stimuli of Experiment 2. ‘*’ Indicate significance for the mixed-effects models. (**C**) Mixed effects model outputs for the Blue and Yellow stimuli of Experiment 3. The ordinate ranges are the same for B and C. The cyan line in B gives the simple model: White = Yellow + Blue, where the Yellow data are those of (**B**) (for 23 subjects) and the Blue data are from (**C**). The cyan line is a reasonable match for the white data of (**B**).

Figure 5 summarises the results for the interaction: ring by attention, for the white, yellow and blue stimuli. The smaller standard errors of the data of Experiment 3 compared to the data for Experiment 1 are indicative of the better signal to noise ratios obtained for the newer stimuli (given similar N). In the case of Experiment 1 none of the attentional effects were significant for the mixed-effects models (Fig. 5A). The rings showing significance for the simpler linear models are marked with a ‘+’. For Experiment 2 the rings of the white stimuli with mean eccentricities of 12 and 24 degrees eccentricity showed significant suppression in the linear mixed-effects models (marked *). The results of Experiments 2 and 3 where similar, with blue stimuli behaving much like the white stimuli. The cyan dotted line of Fig. 5B is the simple sum of the results for yellow in Experiment 2 and those for blue in Experiment 3, on the simple assumption that White = Yellow + Blue, and that the results for experiment 2 provide the best evidence of average behavior for yellow (N = 23).

The results of adjustment for sex and consensual response from these mixed-effects models was very similar across the Experiments, and consistent with previous results. On average, consensual responses were about 94% of direct pupil responses, and females’ responses were about 1.1 times larger on average across the tests, regions etc. We checked for significant regional differences in attentional effects for direct and consensual responses but found none.

**Figure 6:**
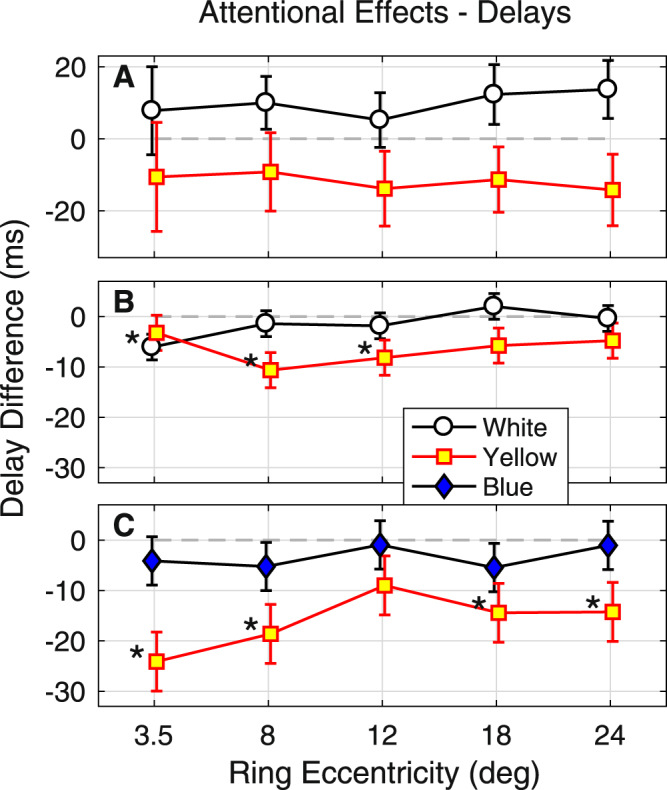
Effects of Attention upon delay as a Function of Eccentricity. (**A**) Experiment 1. There were no significant differences in the time to peak response (delay) for white or yellow stimuli, however yellow stimuli trended towards reduced delay (negative effects). (**B**) For the mixed effects models the white stimuli were faster for the inner ring around the central attentional task (mean eccentricity 3.8 degrees). Yellow stimuli were significantly quicker at 8 to 12 degrees eccentricity. (**C**) For the mixed effects models the yellow stimuli had significantly less delayed responses at 3.5, 8 and 18 degrees eccentricity. Interestingly the effects of centrally directed attention ran in the opposite direction to those for sensitivity (Fig. 5), with yellow stimuli showing larger effects than blue or white.

Figure 6 presents the same analysis as Fig. 5, but where the data are the delays in the time to peak contraction. Surprisingly the results are the reverse of those for pupil amplitude: attention appears to decrease the time to peak (hence the negative differences), but more so for yellow stimuli than for blue or white. For Experiment 2, for which we have the most data, the overall time to peak contraction was 456 ± 59.0 ms (mean ± SD) so the effects are <5% of the total delay. The mean regional t-statistic for delay was 16.8, implying a SE of about 3% of the total delay. Thus, the delays are accurately measured. The significant differences occur both centrally and peripherally, suggesting that perhaps the effects upon delay are more diffusely distributed across the visual field than effects upon sensitivity.

### Experiment 4

To extend these results we investigated the effects of attention directed to peripheral attentional targets placed at ±10 deg eccentricity along the vertical meridian (Methods). Thus, subjects fixated the central cross while attending to the superiorly- or inferiorly-placed target. This was repeated for white and yellow versions of the stimuli in six subjects. Experiments 1 to 3 all compared an Attention and No-Attention cases. The No-Attention case could be thought of as a distributed attention case, although the transient onset stimuli meant that only a few stimuli were presented at any one time (unlike conventional multifocal stimuli where modulating stimulus are continuously presented at all field locations). Nevertheless, it was useful to compare conditions for similarly directed attention. To quantify the effect we computed the difference between the results for superiorly and inferiorly directed attention. These data used the newer Clustered Volleys mfPOP method and the signal quality of these data was higher than the previous experiments providing a median (±SD) t-statistic for response amplitude of 5.53 ± 2.17 for yellow, and 5.58 ± 2.18 for white (cf. Fig. 4).

**Figure 7:**
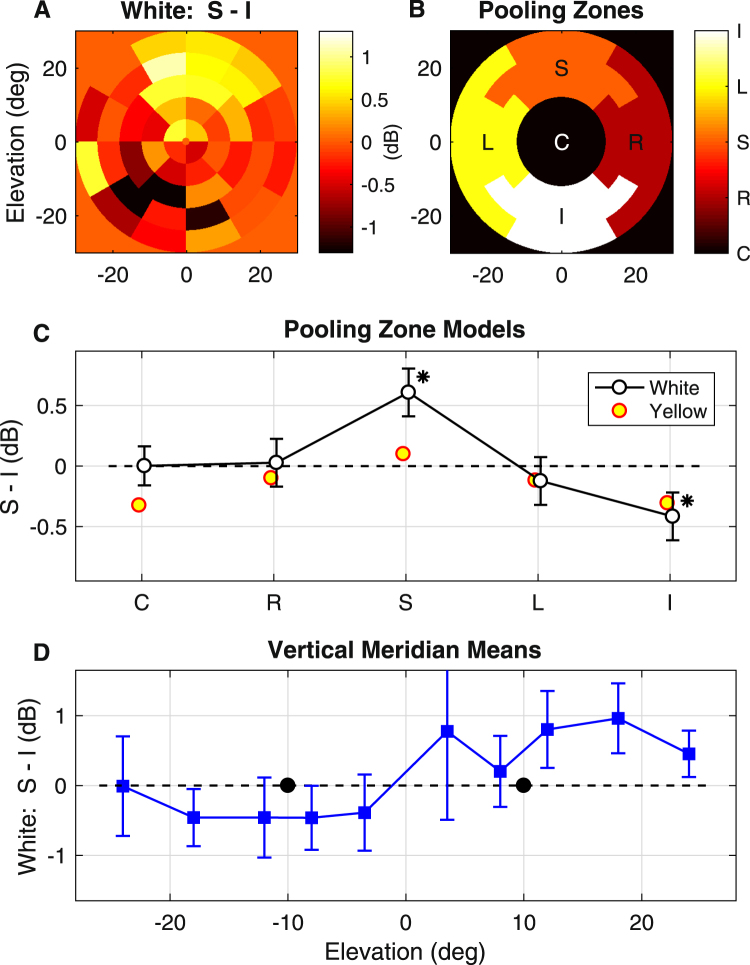
Comparison of direct attention to peripheral targets. Data from Experiment 4. (**A**) Mean sensitivity differences at each location for the differences obtained for white stimuli: Superior – Inferior directed attention (6 subjects). Positive values indicate Superior >Inferior, and negative values Inferior >Superior. (**B**) Illustration of the 5 pooling zones that replaced the 5 rings of earlier eccentricity analyses (Figs 5,6) in otherwise similar linear mixed effects models. (**C**) Results from the mixed effects models showed that only the white stimuli produced significant differences for the Superior and Inferior zones. The * mark the differences that are significant (p < 0.05). The symbols for the white and yellow stimuli are displaced slightly left and right to allow all the dots to be seen. The SE for the yellow data (not shown to reduce clutter) were almost identical to those of for the white stimuli. (**D**) Means of the sensitivity differences (±SE) for test regions straddling the vertical meridian. The position of the attentional targets is given by the black dots at ±10 degrees.

Figure 7A shows the mean difference in sensitivity at each point in the field (computed across eyes, pupils and subjects) for the white stimuli. Thus, positive numbers indicate greater sensitivity where attention was directed to the superior field and negative values indicate greater sensitivity where attention was directed to the inferior field. There are clear effects superiorly and more diffuse effects inferiorly. Figure 7B indicates the 5 zones used to pool (average) results for the mixed effects models, meaning these models parallel the 5-ring models of the previous experiments. Figure 7C shows the results from the models for white and yellow stimuli. Only the white stimuli provided significant results for the superior (S) and inferior (I) zones, 0.61 ± 0.19 dB and −0.42 ± 0.19 dB (mean ± SE) respectively. Figure 7D shows means of the pairs of regions straddling the vertical meridian of the field (±SE). The position of the attentional targets are indicted by black circular dots. It appears that the attentional effects are fairly diffuse and appear to extend to more peripheral locations. Examining the regions of the left and right zones of Fig. 7A indicates some suppression away from the point of attention. Analysis of response delays showed no significant differences.

## Discussion

The main finding of this study was that attention to the central visual field tended to produce suppression of sensitivity away from that focus. This is similar to the findings of fMRI studies that have employed multiple, concurrently presented stimuli across the visual field^17,18,21^. A point of difference was that stimuli containing blue (white stimuli) seemed to show stronger effects than yellow stimuli. To our knowledge, no such colour differences have been explored using fMRI. Conversely, attention to yellow stimuli accelerated pupil responses, while those to blue or white stimuli had little to no effect on delay. We are unsure as to why these delay effects occur but their presence strengthens the case that something different is occurring for stimuli that do, or do not contain blue. When the effects of peripheral attention were examined (Fig. 7) the observation was mainly of increased responses in the vicinity of the attended target, which was similar to the largest fMRI study of the effect of peripheral attentional targets^21^. Again effects were larger for white stimuli than yellow.

Although the responses reported here were well measured (Fig. 4) there appeared to be some variation across the field of the effects of attention (Figs 3C,F, 5 and 6). Of course, we do not yet know the details of how the effects of attention actually vary across the field, or across subjects. This is especially the case for the natural situation where all parts of the whole field are stimulated in parallel, all at the same time. What we do know mainly comes from three fMRI studies that used multifocal stimulus arrays that covered a similar portion of the fields as used here. One fitted attentional effects as difference of Gaussians (DOG) for the data of 4 subjects. In that study the point of attention followed a circular path at a fixed eccentricity during testing. Thus, the measured “fields” were the average for the circular set of attentional foci. That method allowed the broader effects of suppression by attention to be identified in higher cortical areas, some of which supply the pupils^30^. A second study showed high variability across its five subjects^17^. The third examined the effects of attention at 4 eccentric foci in 19 subjects, and found that attentional suppression was variable and strongest in the two superior quadrants of the visual field, while enhancement was greatest in the inferior field.^21^. As here the effects were quite diffuse and extended quite far peripherally. Mind you the BOLD changes observed were on the order of 0.5% rather than the 15% observed here (e.g. 0.6 dB, Fig. 7). None of these studies used dichoptic stimuli, nor did they investigate colour as used here. Overall new experiments and better methods are needed to understand the details of the distribution of attentional effects across the field. Our new Clustered Volleys stimuli include macular versions, which may prove interesting given the recent demonstration effects of directed attention within the fovea.^34^ Also they could allow more direct comparison with the fMRI studies of Simola *et al*.^21^ that sampled the central 20 degrees, with peripheral attentional targets presented at 4 degrees.

Colour dependent attentional effects are not totally without precedent. Work by Morrone *et al*.^35^ indicates that greater attentional effects occur when the chromatic properties of centrally attended targets match those of the peripheral stimuli. Here the red content of the peripheral stimuli and attentional tasks was similar except for the blue stimuli of Experiment 3.

Attentional effects are thought to be mediated by higher cortical areas exerting top-down influences^36–38^, which in humans not only extend to occipital cortical areas^39^, but also to modulation of the lateral geniculate nucleus (LGN)^40^, and the superior colliculus^41–43^. Thus, our results need to be discussed in terms of the blue sensing system and thalamo-cortical connectivity to the pupillary system of its visual inputs.

The pretectal olivary nuclei (PON) drive the pupils and receive about half their input directly from retinal ganglion cells (RGCs), and the remainder from brain areas including the striate and extra-striate cortex^30^. Perhaps the best-known cortical input to the pupils is the luminance-based *accommodative triad*, which converges the eyes, accommodates the lenses and constricts the pupils when objects draw near^44^. This cortically derived, stereopsis-driven, system also appears to make the pupils sensitive to equiluminant temporal modulation of very high spatial frequency luminance gratings^45^. Pupil responses to red-green equiluminant stimuli, can be reversibly modulated from macaque extrastriate cortex^46^, or blocked by related cortical lesions in humans^47^. Those patients display red-green achromatopsia, thus the cortically mediated visual sensation and the pupil responses are concordant. As we have confirmed with 44-region multifocal stimuli^31^, these cortically driven pupil responses to red-green equiluminant stimuli are delayed compared to responses to luminance stimuli^48^. Pupil responses to 13 Hz modulation of blue-yellow contrast are similarly delayed^49^.

The blue content of white stimuli has been reported to be a major source of between-subject variability in multifocal ERGs^50^ due to the different blue-blocking capacity of brunescent lenses in older persons. The subjects in the present study were too young for this to be a major factor. At the level of the retinal ganglion cells (RGCs) the blue system is quite heterodox compared to other RGC types. In primates, the luminance and red/green RGCs come in anatomically similar pairs of parasol and midget RGCs, with the inputs to their receptive field centres exchanged to provide ON or OFF luminance centres, and red-ON or green-ON centres. The anatomically similar members of each pair of RGC types differ principally in which strata of the inner plexiform layer (IPL) their dendrites reside^51^. By contrast, the blue-ON and blue-OFF signals we experience are gathered by anatomically very different sub-populations of RGCs. The *small-bistratified* RGCS carry the cone-driven Blue-ON/Yellow-OFF signal, while the cone-driven blue-OFF/Yellow-ON signal is transmitted by the *giant-monostratified* RGCs^52^. These cells are much larger than the bistratified RGCs and have sub-populations defined by their stratification in the IPL and whether their axons project to the LGN or the PON.

All the *giant-monostratified* RGCs contain melanopsin, making them *intrinsically photo-receptive* to blue light^52^, and so they are sometimes referred to as ipRGCs. The melanopsin has no light adaptation mechanism, which gives the ipRGCs a Blue-ON response that tracks absolute luminance in bright conditions^53^. The melanopsin response is very sluggish and is the main determinant of the steady state pupil diameter in bright light^53–55^. The cone derived Blue-OFF and melanopsin derived Blue-ON responses are thus temporally duplexed. Thus, post-synaptic cells could largely select which blue signal they are attending to (ON or OFF) by either integrating with a time constant of about 1 second, or by differentiating the input to monitor frequencies above 1 Hz.

The key factor for the present study is that both types of blue sensitive RGCs appear to contribute to visual perception via their projections to the LGN. As summarised above these signals then return to the pupillary system via the extra-striate cortical input to the PON^30^. That multifocal stimuli such as those used here influence the pupils via this route has been recently demonstrated by several of our studies. We have compared yellow and red-green stimuli in healthy subjects^31^ and in glaucoma patients^23^. As shown by other studies the cortically derived pupil responses to red-green stimuli were delayed compared to luminance stimuli. Both stimulus types picked up visual field losses in glaucoma suggesting the pupillary responses to these transiently presented stimuli are concordant with visual perception. By contrast, when extremely slow, long duration, blue stimuli that drove melanopsin were used the ability to detect glaucomatous visual field loss was poor compared to our standard transiently presented yellow stimuli^24^. That may be because of the very large dendritic trees of the ipRGCs or the branching of ipRGC axons within the retina^56^. Overall, it seems we can at least partially choose cortical versus retinal sources by altering the temporal properties of the stimuli. This idea is supported by our demonstration that a visual field marker for early-stage macular degeneration and diabetic eye disease is seen both for yellow mfPOP and mfVEP testing on the same 47 patients when the mfVEP electrodes are positioned to pick up extrastriate cortical activity^32^. In summary, several sources of evidence indicate that mfPOP responses to transiently presented stimuli are substantially driven by the extrastriate cortex, as PON connectivity would suggest^30^.

Reports of the ability of the pupils to track aspects of attention and cognition have been growing, but so far not with a detailed spatial mapping of the attentional affects as shown here. For example, pupil constrictions to dim photographs of the sun have been reported^57,58^. Similarly, constriction and dilation effects have been shown with respect to modulations of attention and the apparent brightness of stimuli^59,60^. Attention, but not day-dreaming, modulates pupil dilation mediated by the slow sympathetic nervous system^61^. Here we have concentrated on the higher-bandwidth pupil constriction system driven by the parasympathetic system. That being said we have demonstrated that mfPOP can quantify the temporal evolution of the sympathetic-driven re-dilation phase out to several seconds^24^. Those parts of the pupillary response have been shown to quantify very interesting aspects of attentional modulation^60,61^. Thus, these effects could also be assessed in a multifocal setting, and differences between contraction and dilation effects quantified in one test.

The multifocal methods used here open up the possibility of examining retinotopic mechanisms of attention as has begun to be explored with fMRI^17,18,21^, but with a cheaper and more user-friendly apparatus. One could also combine pupillometry and fMRI, or pupillometry, fMRI, and Near Infrared Spectroscopy. As we have recently demonstrated mfVEPs measured using EEG electrodes that largely sample extra-striate visual cortex verify some aspects of mfPOP responses^33^, and there would be no reason not to do both simultaneously to better identify the sources of pupil responses to attention. Certainly, the set-up for mfPOP is shorter than for EEG, and the system is potentially very portable. The dichoptic set-up also allows for a variety of between-eye stimulus manipulations. An aspect we have not addressed is that by stimulating both eyes concurrently and recording both pupils both direct and consensual responses are recorded. This allows for differential examination of afferent versus efferent effects of attention. Some of our results indicate that efferent effects were large but confirmation will await the use of newer more reliable stimuli^26^.

Perhaps the rate of delivery of multifocal stimuli needs to be considered more. Differences between Experiment 1 and the other three were observed, and the stimulus delivery rate in Experiment 1 was 4 times higher. Aside from the stimuli being different, an idea suggested by a reviewer is that with the more rapid stimuli attention would possibly be more divided between them, when there was no central task.

A problem with the first three experiments was the possibility that the participants either guessed the purpose of the experiment, or invented some attentional task of their own during the experiments, when they were not directed to attend to the central changes. It was therefore useful to compare results obtained for different foci of continuously directed attention as in Experiment 4 (Fig. 7). Nevertheless, it was reasonable to attempt the Attention vs. No-Attention case in the first instance and that is what the closest fMRI studies have used^21^. Experiment 4 examined differences between different direct attention conditions and revealed that attention in the inferior field may be more variable.
